# Quality assessment with diverse studies (QuADS): an appraisal tool for methodological and reporting quality in systematic reviews of mixed- or multi-method studies

**DOI:** 10.1186/s12913-021-06122-y

**Published:** 2021-02-15

**Authors:** Reema Harrison, Benjamin Jones, Peter Gardner, Rebecca Lawton

**Affiliations:** 1 School of Population Health, UNSW Sydney, Sydney, Australia; 2 School of Pharmacy and Medical Sciences, University of Bradford, Bradford, UK; 3 Institute of Psychological Sciences, University of Leeds, Leeds, UK

**Keywords:** Quality appraisal, Mixed-methods research, Multi-methods research, Systematic review, Health services research

## Abstract

**Background:**

In the context of the volume of mixed- and multi-methods studies in health services research, the present study sought to develop an appraisal tool to determine the methodological and reporting quality of such studies when included in systematic reviews. Evaluative evidence regarding the design and use of our existing Quality Assessment Tool for Studies with Diverse Designs (QATSDD) was synthesised to enhance and refine it for application across health services research.

**Methods:**

Secondary data were collected through a literature review of all articles identified using Google Scholar that had cited the QATSDD tool from its inception in 2012 to December 2019. First authors of all papers that had cited the QATSDD (*n*=197) were also invited to provide further evaluative data via a qualitative online survey. Evaluative findings from the survey and literature review were synthesised narratively and these data used to identify areas requiring refinement. The refined tool was subject to inter-rater reliability, face and content validity analyses.

**Results:**

Key limitations of the QATSDD tool identified related to a lack of clarity regarding scope of use of the tool and in the ease of application of criteria beyond experimental psychological research. The Quality Appraisal for Diverse Studies (QuADS) tool emerged as a revised tool to address the limitations of the QATSDD. The QuADS tool demonstrated substantial inter-rater reliability (k=0.66), face and content validity for application in systematic reviews with mixed, or multi-methods health services research.

**Conclusion:**

Our findings highlight the perceived value of appraisal tools to determine the methodological and reporting quality of studies in reviews that include heterogeneous studies. The QuADS tool demonstrates strong reliability and ease of use for application to multi or mixed-methods health services research.

**Supplementary Information:**

The online version contains supplementary material available at 10.1186/s12913-021-06122-y.

## What is known

- Many tools exist for assessing the quality of studies in systematic reviews of either quantitative or qualitative work.
- There is a paucity of tools that assess the quality of studies within systematic reviews that include a diverse group of study designs, and mixed or multi-methods studies in particular.
- The Quality Assessment Tool for Studies with Diverse Designs (QATSDD) published in 2012 was developed to assess the quality of studies with heterogenous designs primarily for use in the discipline of Psychology.

## What this study adds

- The Quality Assessment for Diverse Studies (QuADS) tool is a refined version of the QATSDD tool. The aim was to use survey and literature review data to enhance the applicability of the tool to health services research, and more specifically, to multi or mixed-methods research.
- The QuADS tool demonstrates substantial inter-rater reliability and content and face validity.

## Background

The inclusion of diverse types of evidence, such as qualitative and mixed or multi-methods research, is well-established in systematic reviews of health services research [1–3]. This is important because these methods can address the complexities within healthcare that cannot often be readily measured through a single method. Qualitative methods, when used alone, offer explanatory power to enhance understanding of multi-faceted and complex phenomena such as experiences of healthcare and systems [3]. When partnered with quantitative methods, qualitative data can support and add depth of understanding [4, 5].

The appraisal of the methodological quality, evidence quality and quality of reporting of individual studies and of studies included in a review collectively is firmly established for reviews of quantitative studies. There are more than 60 tools currently available to assess the quality of randomised controlled trials alone [6]. Appraisal of the quality of evidence is often used to assess bias, particularly in randomised controlled trials. More recently, quality appraisal tools have extended to tools for appraising qualitative research, with the emergence of multiple tools in this space [7] creating a topic of extended debate [7–10]. As a result, reviews that include both qualitative and quantitative research often utilise separate quality appraisal tools for the quantitative and qualitative studies within the review, often citing the lack of a standard, empirically grounded tools suitable to assess methodological quality, evidence quality and/or quality of reporting with a variety of study designs [11]. The use of a parallel approach to all aspects of quality appraisal offers strength in the ability to acknowledge the unique nature of qualitative research and its epistemological distinction from quantitative approaches. Yet, a dual approach does not facilitate the appraisal of methodological, evidence or reporting quality for mixed-methods research, and creates challenges in appraising these aspects of multi-methods work.

Thus, acknowledging that the underlying assumptions of quantitative and qualitative research are substantially different, a tool to appraise methodological quality, evidence quality and/or quality of reporting mixed- or multi-methods research is valuable in enabling researchers to consider the transparency and reporting of key elements of these approaches [12]. Moreover, a tool that is relevant to mixed- and multi-method approaches is significant in the context of growing recognition of the value of these methodologies in health systems and services research [4]. A single tool that can be used to evaluate methodological quality, evidence quality, and quality of reporting across a body of diverse evidence facilitates reviewers to reflect on the extent to which there is apparent transparency and congruency in the research purpose and its reporting and the implications for evidence quality. This is currently not available for mixed- and multi-methods work, with study heterogeneity as a key obstacle to evidence appraisal. Given the complexities of multiple individuals evaluating a diverse set of studies, a supporting tool may also provide an underpinning method to develop a shared understanding of what constitutes quality in research methods, evidence and reporting.

The authors published in 2012, a pragmatic approach to facilitate reviewers to appraise the methodological quality, evidence quality, and quality of reporting in reviews that included qualitative, quantitative, mixed- and multi-methods research using a single tool (QATSDD) [13]. The QATSDD has been cited more than 270 times to date and has been used in more than 80 reviews. The tool provides a framework for exploring the congruency, transparency and organised reporting of the research process for research grounded in post-positivistic or positivist methodology that informs multiple-methods or mixed-methods designs. The tool was not proposed as a basis for determining studies to be excluded from a review given that any cut-off points to indicate high or low quality would be arbitrary.

The QATSDD tool was originally developed for application in Psychology but has demonstrated wider relevance through its application in a broad range of health services research. Its wide use suggests that researchers value the ability to appraise quality of evidence from studies that employ or combine a range of methods. Yet the QATSDD tool has some limitations in its ease of use beyond the discipline of Psychology. We therefore aimed to revise, enhance and adapt the current QATSDD tool into an updated version; Quality Assessment for Diverse Studies (QuADS), for greater applicability to health services researchers appraising quality of methods, evidence and reporting in multi- and/or mixed-methods research.

## Methods

### Data sources and procedures

Studies citing the QATSDD tool were identified using Google Scholar, citations imported to the reference-management software (Endnote X9.2) and duplicates removed. Full-text screening of the identified studies and discussion between two authors (BJ and RH) was used to identify studies that included qualitative evaluative data or commentary regarding the QATSDD tool to inform its enhancement. The following data were extracted: first author, year of publication, country, research discipline, study synopsis, QATSDD reliability and validity data and qualitative evaluative comments about the use of the tool. Alongside the review of citing studies, all authors who had used the QATSDD in a published, publicly accessible paper (101 authors) were contacted to provide an opportunity for them to provide any additional feedback through a qualitative brief online survey form. Ethical approval to administer the survey form was granted from the UNSW Human Research Ethics Committee (HC190645). The survey form contained two open-ended, free-text response items: 1) ‘When applying the QATSDD in your research, what were the strengths of the tool and what did this enable you to achieve?’ and 2) ‘When applying the QATSDD in your research, what were the limitations or challenges you experienced and how could these be addressed in a revised version of the tool?’ The survey was administered by one author (BJ) to the email addresses of the study authors via the Qualtrics online survey software, with one follow-up reminder. Consent was implied through completion and submission of the survey form.

### Data analysis and synthesis

A narrative synthesis [14] was then undertaken with the heterogeneous data emerging from the literature review in addition to the qualitative comments provided by the survey respondents. In the development of the primary synthesis, two authors (BJ, RH) independently undertook a line-by-line review of each study and survey content. The evaluative comments were labelled and merged into a table of the items arising. The authors then met to discuss the commonly occurring items and created initial themes. In a further stage, an exploration of the relationships in the data and an assessment of the robustness of the synthesis product was explored. The initial themes were discussed and refined with two further authors (RL, PH) into final themes, which were tabulated. The research team then collectively discussed areas for clarification and areas requiring changes to be made. An iterative process of making refinements to the tool drawing upon the synthesised data was undertaken through collaboration, review of the tool and discussion between the author team.

### Preliminary internal assessment and external evaluation

Face and content validity were also explored through providing the revised QuADS tool to 10 researchers who had expertise in reviewing studies with diverse designs within systematic reviews. The researchers worked across different disciplines (psychology, sociology, health services research, pharmacy) and methodologies (quantitative, qualitative and mixed-methods) relevant to health in the UK or Australia. Each researcher was provided the tool via email and asked to 1) provide their feedback on the perceived suitability of the items within the tool to their own field and methods of research and 2) report any items that require clarification for ease of use or readability. Their feedback was discussed between the authors and used to revise the tool iteratively through a series of minor amendments to wording and ordering or the tool items. The resulting QuADS tool was also subject to inter-rater reliability analysis between a psychologist, public health and health services researcher through application to 40 studies a recent systematic review with a kappa of 0.65 published by a colleague within our department who was external to this study [15].

## Results

### Results of the review

**Fig. 1:**
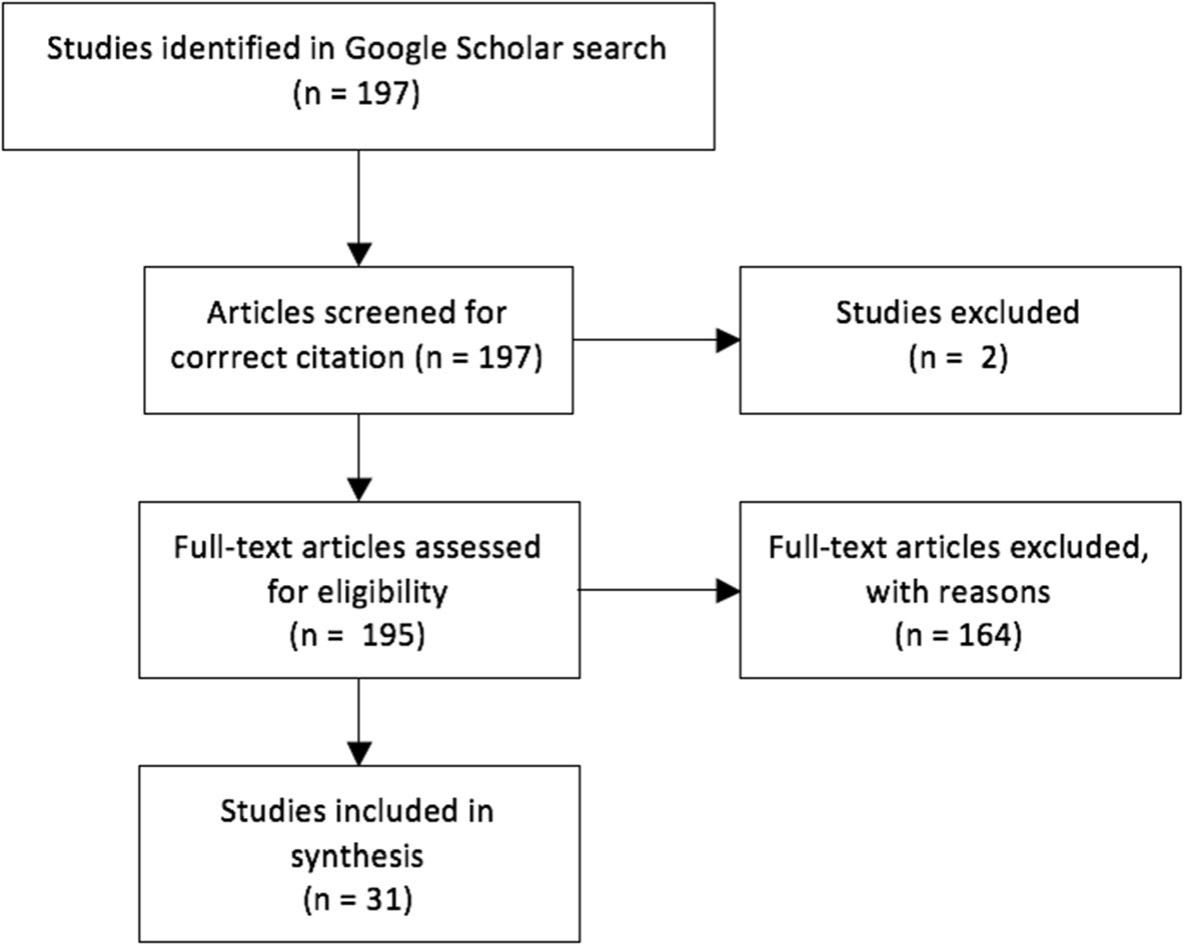
Flow chart of study search and selection process

One hundred and ninety-seven citations were attributed to Sirriyeh et al’s (2012) [13] *Reviewing studies with diverse designs: the development and evaluation of a new tool* article and 31 of these studies met the inclusion criteria by including evaluative data or comments (Table 1). The study selection process is shown in Fig. 1. Of the 101 authors who had cited the QATSDD paper and reported using the tool in their publication; 13 did not receive emails, 10 had moved institutions or were on leave,, 74 did not provide any additional feedback and 1 researcher replied stating they had not been the individual that had used the tool. Three respondents provided survey feedback which were synthesised with and aligned the findings from the reviewed articles.

**Table 1:** Summary of Included Studies

First author	Year	Country	Discipline	Synopsis of review	Reliability and validity	Evaluative comments
Abda A [16].	2018	Canada	Psychology	A systematic review that investigated the psychosocial outcomes of children and adolescents with severe congenital heart disease.	This tool was selected for its good inter-rater reliability (k ¼ 71.5%) and its ability to highlight the methodological strengths and weaknesses of studies (Sirriyeh et al., 2012).	-
Adam A [17].	2016	Denmark	Health Sc	A systematic review that investigated the effectiveness of obesity related interventions at retail grocery stores and supermarkets.		Transparent and validated tool.
Albutt A.K [18].	2016	UK	Psychology	A systematic review that investigated the role of patients and their relatives in escalating clinical deteriotation in hospital settings.	Strong and significant correlation between 1st and 2nd reviewer’s quality assessments, r = .73,P.039.	
Alsawy S [19].	2017	UK	Psychology	A mixed-methods systematic review that investigated what good communication is for people living with dementia.	Agreement of 96.0 and 94.4% was achieved between the first researcher (SA) and independent raters 1 and 2 respectively. Statistically significant (*p* < 0.00) inter-rater reliability of quality assessments was achieved across all three raters (the first researcher and two independent).	The outcomes suggest overall agreement in the quality ratings of each study and robustness of the QATSDD tool.
Arbour-Nicitopoulos K.P [20].	2018	Canada	Allied Health	A scoping review investigating the inclusive out-of-school time Physical activity programs for children and youth with physical disabilities.	This quality assessment tool has demonstrated good face validity and interrater and test–retest reliability for examining study quality across diverse methodologies.	Three reviewers independently appraised the quality of each of the included studies (53.5% agreement) using a 16-item quality assessment tool that can be applied to quantitative, qualitative, and mixed-method studies. Consensus was obtained on rating discrepancies through group discussion. - Selection of this tool was based on its consideration of additional elements that are often not taken into account in study quality yet are critical to external validity of the study findings e.g. application of theoretical frameworks and/or constructs to the research, evidence of user involvement in the study design and discussion of strengths and limitations.
Augestad L.B [21].	2017	Norway	Psychology	A systematic review to investigate self-concept and self-esteem among children and young adults with visual impairment.	The tool, which was developed to assess the quality of studies on one topic but using different approaches or designs, has been found to have good reliability and validity (Sirriyeh et al., 2012).	-
Augestad L.B [22].	2017	Norway	Medicine	A systematic review to investigate mental health among children and young adults with visual impairments.	The tool has been found to have good reliability (Cohen’s kappa, 71.5) and good face validity (Sirriyeh et al., 2011). - The weighted kappa was 0.5 (indicating moderate agreement), and the Spearman correlation was 0.75 (indicating a strong association or relationship).	-
Aztlan-James E.A [23].	2018	America	Medicine	A systematic review investigating multiple unintended pregnancies in U.S. women.	The validity and reliability of QATSDD is established and has been reported. In case of disagreements, the study was discussed until agreement was reached on quality score.	-
Band R [24].	2015	UK	Psychology	A systematic review investigating patient outcomes in assosication with significant other responses to chronic fatigue syndrome.	The measure has demonstrated adequate reliability (Sirriyeh et al., 2012), although normative values associated with study quality are not currently available.	-
Batten G [25].	2014	UK	Psychology	A systematic review investigating the factors associated with social interactions between deaf children and their hearing peers.	Inter-rater reliability scored at 0.743(using Spearman’s Correlation) at < 0.01 significance level(for a 43- question devised checklist combining QATSDD with 3 other QA checklists).	
Baxter R [26].	2016	UK	Health Sc	A systematic review investigating the methods used to apply positive deviance within healthcare organisations.	Validated tool that standardises the quality assessment of research with heterogenous study-designs.	
Blackwell J.E [27].	2017	UK	Psychology	A systematic review investigating the cognitive function and psychosocial well-being in school-age children with narcolepsy.	Substantial inter-rater agreement (89.3%),remaining differences solved by discussion.	Particularly suited as QATSDD involves qual and quant aspects both.
Blake D.F [28].	2018	Australia, New Zealand, Canada	Medicine	A systematic review investigating the effects of helicopter retrieval on injured divers.		Studies identified were of diverse designs so the modified QATSDD tool was used to better compare the levels of evidence.
Bradford N [29].	2019	Australia	Nursing	An integrative review investigating the evaluation frameworks in health services.	-	We added a fifth item—(Not Applicable) for articles that were narrative discussions rather than research per se. Two authors (NB and SC) independently appraised the included articles with high agreement (92%). Despite the QATSDD being designed for disparate study designs, many criteria were not applicable to the type of papers included in this review.
Braun S.E [30].	2019	USA	Psychology	An integrative review investigating mindfulness in health care professionals and its relation to patient care.	-	Although this tool was not developed to evaluate cross-sectional studies, it can easily be applied to cross-sectional designs without omitting domains or adapting the tool; furthermore, it has been used in previous systematic reviews to assess cross-sectional research.
Burton A [31].	2016	UK	Psychology	A systematic review and meta-analysis investigating mindfulness-based interventions for reducing stress among healthcare professionals.		QATSDD combines previously validated tools to produce a comprehensive list of indicators of good quality research.
Carrara A [32].	2018	Switzerland	Health Sc	A systematic review investigating the role of health literacy in predicting adherence to nutritional recommendations.	QATSDD has shown good reliability and validity in evaluating the quality of methodologically diverse studies in the contexts of psychology, sociology and nursing.	
Clausen C [33].	2017	Canada	Health Sc	An integrative review investigating educational interventions that enhance competencies for interprofessional collaboration among nurse and physician managers.	Quality Assessment Tool (QAT) tool was chosen for quality appraisal of the included studies. This tool, tested by the authors for reliability and validity, was chosen for its rigor in the assessment of qualitative, quantitative, and mixed method studies.	Although the QAT tool was transferable across studies, all qualitative articles scored poorly. One could question whether the tool was well adapted and reliable to provide sufficient comparison amongst studies.
Connolly F [34].	2017	Ireland	Health Sc	A systematic review of the barriers and facilitators related to the implementation of a physiological track and trigger system.	Validated tool for assessing study quality.	Inconsistencies in scoring were resolved through discussion.
Curran C [35].	2018	Ireland	Medicine	A systematic review investigating the primary care safety climate survey instruments.		This tool allows standardized evaluation of studies with varying research designs.
Deming A [36].	2019	US	Health Sc	A study investigating the absence of evidence-based practices (EBPs) in the treatment of sexual abusers.		Several systems for scoring and rating research studies of diverse designs and methodologies have been developed and described (including QATSDD). Each approach recommends somewhat different methods or systems for determining the overall strength of research, but none have been developed specifically for use with research relating to individuals with a history of sexual offending.
Dias C.C [37].	2013	Portugal	Medicine	A systematic review and meta-analysis investigating the clinical prognostic factors for disabling Crohn’s disease.		Allows comparison of different study designs
Emerson L.M [38].	2017	UK	Psychology	A systematic review and narrative synthesis investigating the teaching of mindfulness to teachers.		Additional Item “Clarity of Intervention” added to QATSDD, initial agreement between the researchers was 91.6%, calculated using Cohen’s Kappa. Discrepancies were resolved through discussion.
Fenton L. [39]	2016	Canada	Health Sc	An integrative review investigating the benefits of recreation for the recovery and social inclusion of individuals with mental illness.	QATSDD has been evaluated for validity & reliability.	Only tool specifically designed to evaluate diverse research approaches.
Fenton L. [12]	2015	Canada	Health Sc	A comments and critiques paper investigating the QATSDD critical appraisal tool.		Potential value but a number of aspects for clarification: -Unclear meaning of language -Further definition of language in each indicator and inclusion of explicit examples for each criterion recommended - Needs outlining of clear parameters around the use of tool, stating that the tool should be used in synthesis work for studies of mixed methods or work that includes qual and quant research informed by a positivist paradigm - Tool is subjective in nature - Dropping the scoring system recommended - “Evidence of User Involvement in design” is inappropriate - No indicator addressing bias included
Filmer T [40].	2018	Germany	Medical Education /Medicine	A systematic review investigating the effectiveness of interventions teaching cross-cultural competencies to health-related professionals with work experience.	- For all criteria ratings, the unadjusted two-way random single-measure intraclass correlation coefficient (2,1) was 0.93, confirming a very good reliability.	Any discrepancies in ratings were discussed and a consensus was achieved.
Fylan, B [41].	2015	UK	Health Sc	A thesis paper that investigated medicines management after hospital discharge.		It was chosen as a suitable tool because of the heterogeneity of research designs in the literature
Graham-Clarke E [42].	2018	UK	Psychology	A systematic review and thematic synthesis investigating the facilitators and barriers to non-medical prescribing.	A validated quality-assessment tool.	Two reviewers independently assessed the studies using the tool; resolving any disagreement in the scores through discussion
Gillham R [43].	2015	UK	Psychology	A systematic review investigating the outcomes for women admitted to a mother and baby unit.	Interrater reliability was very good (k = 0.91).	
Gkika S [44].	2017	UK	Psychology	A systematic review investigating social cognition and metacognition.		To examine potential assessment bias,25% of papers were independently assessed by a colleague and good agreement between 2 raters was observed.
Hardy M [45].	2016	UK	Medicine	A systematic review investigating if radiography advanced practice improves patient outcomes and health service quality.	High interrater agreement (K = 0.89).	Components fulfil the criteria for quality assessment within “Centre of Reviews and Dissemination Guidance”.
Harris K [46].	2016	UK	Psychology	A systematic review investigating distress in significant others of patients with chronic fatigue syndrome.		There may be disadvantages of using a quality assessment tool scored from 0 to 3 as opposed to a dichotomous (yes/no) rating scale. One potential disadvantage is that a greater number of response options in the scale may increase the subjectivity of the ratings. In contrast, a dichotomous scale could have been rated on the absence or presence of key information, which would have provided fewer opportunities for bias. The study design should be taken into account when interpreting the study’s findings.
Harrison R [47].	2015	Australia	Health Sc	A systematic review investigating the patient safety and quality of care in developing countries in Southeast Asia.	Disagreements were resolved through discussion resulting in substantial agreement (k = 65.8%) on a random sample of 30% papers.	
Harrison R [48].	2015	Australia	Health Sc	A systematic review investigating patients’ experiences of adverse events in health care.	Disagreements between 2 reviewers resolved by discussion resulting in substantial agreement (k = 61.6%).	
Harrison R [49].	2014	UK	Nursing	A narrative review investigating the contribution of nurses to incident disclosure.	Disagreements between reviewers resolved through discussion and substantial agreement (k = 73.7%) reached.	
Hawkins R.D [50].	2017	UK	Social Sc.	A systematic review investigating the psychological risk factors for childhood nonhuman animal cruelty.	The publications were scored by 2 authors independently (x = 0.78),with Cohen’s kappa demonstrating a substantial strength of agreement.	Case studies could not be easily assessed using these criteria.
Heath G [51].	2016	UK	Health Sc	A mixed-methods study that investigated the development of a tool that support communication of parental concerns when a child is in hospital.	The QATSDD has in a preliminary assessment been shown to have good face validity, as well as good interrater and test-retest reliability in evaluating qualitative as well as quantitative studies.	-
Hesselstrand M [52].	2015	Sweden	Allied Health	A systematic review investigating occupational therapy interventions in chronic pain.	The QATSDD has in a preliminary assessment been shown to have good face validity, as well as good inter‐rater and test–retest reliability in evaluating qualitative as well as quantitative studies (Sirrieyh et al., 2012)	-
Hill S [53].	2015	UK	Health Sc	A rapid review investigating the conduction of contingent valuation studies in older and young populations.		Quality assessment was considered; however following examination of potential tools available for the process, a decision was made not to progress with quality assessment. Although this review included studies of diverse design, it was felt that the items in the tool devised by Sirriyeh et al. (2012) did not allow sufficient focus on the methods used (i.e. contingent valuation)
Holl M [54].	2015	Netherlands	Social Sc	A systmetic review investigating the interventions to prevent tenant evictions.	Weighted kappa was 0.70 (substantial agreement)	QATSDD does not provide cut-off points for quality rating of individual studies as good, fair or poor.
Iddon J.E [55].	2016	UK	Psychology	A systematic review investigating positive psychological interventions and chronic non-cancer pain.	QATSDD has shown good reliability and validity when assessing the risk of bias and quality of diverse study designs.	Although there are implications and subsequent limitations to consider when applying a more general tool for quality assessment,the QATSDD allowed for cross-comparison between differing methodologies. Whilst this has its advantages, QATSDD total scores should be interpreted with some discretion as particular areas of significant methodological weaknesses may be concealed by perhaps less influential strengths in other areas, and vice versa. For example, a poor score on the item assessing the appropriateness of the study sample size may be obscured by a higher score merited from describing the study research setting in detail.
Jaarsma E.A [56].	2018	UK	Psychology	A systematic review investigating the promotion of physical activity for disabled people who are ready to become physically active.		Used with the exception of Criteria 14 (Reliability of analytical process-qualitative only) because this check is flawed and now known to be ineffective for reliability purposes in terms of qualitative research (Smith & McGannon 2017)***, all included studies were assessed for quality based on the tool by Sirriyeh et.al.
Jackman P [57].	2019	England	Health Sc	A systematic review investigating flow states in exercise.	-	Used with the exception of criterion 14, which was omitted due to recent criticism of reliability strategies for qualitative research (Smith & McGannon, 2018). - During these critical discussions, the authors highlighted some concerns with the study quality scores as the QATSDD was unable to detect many of the conceptual and methodological issues identified by this review
Jackson-Blott K [58].	2019	UK	Psychology	A narrative literature review investigating recovery-oriented training programmes for mental health professionals.	An inter-rater reliability of 71% was obtained between reviewers (two of the authors) on a random sample of four papers (25%).	The scoring system of methodological quality (QATSDD) accounted for the diversity of study designs and inter-rater reliability checks provided assurance of its rigorous application.
Johnson D [59].	2017	Australia	Psychology	A systematic review investigatingthe resilience to emotional distress in response to failure, error or mistakes.		QATSDD used to assist in the development of coding matrix for this study using “iterative” process.
Jones N [60].	2018	UK	Health Sc	A systematic review investigating visual impairment on nutritional status.	QAT has been reported to have good validity and reliability.	
Khajehaminian M.R [61].	2018	Iran	Health Sc	A systematic review investigating the criteria and models for casualty distribution in trauma related mass casualty incidents.		In the case of quality appraisal of the included literature an extensive search to find appropriate tool was unsuccessful. Although there were some tools for appraising diverse design studies, [including QATSDD], they are applicable for medical studies. In this case, there is a need to develop a new quality appraisal tool to assess studies with diverse design in a multidisciplinary research environment.
Klingenberg O [62].	2019	Norway	Social Sc	A systematic review investigating digital learning in mathematics for students with severe visual impairment.	-	For example, it is not defined or clearly explained how the indicators ‘theoretical framework’ or ‘the statement of aims’ should be scored, which may imply a change in construct validity. The QATSDD should therefore be used with caution. Studies scoring above 75% were considered ‘high quality’, 50–75% ‘good quality’, 25–50% ‘moderate quality’, and those scoring below 25% ‘poor quality’.
Kolbe A.R [63].	2015	Haiti, USA	Social Sciences	A qualitative study investigating transactional sex between UN peacekeepers and Haitian citizens.	-	‘‘Redundancy” as ideal when little existing data available about the extent of phenomenon.
Kumar M.B [64].	2012	Canada	Health Sc	A literature review investigating trends in Métis-related health research from 1980 to 2009.	Inter-rater agreement was substantial (k = 0.67).	-
Lambe, K [65].	2019	Ireland	Patient Safety	A systematic review investigating hand hygiene compliance in the ICU.	The tool has been assessed by its authors for reliability and validity and was used by two authors for each study in this review.	The evaluation is subjective and concerns have been raised about the tool’s structure, particularly around the equal weighting of all items for all studies.
Lamore K [66].	2017	France	Psychology	A systematic review investigating treatment decision-making in chronic diseases and family members’ roles, needs and attitudes.	QATSDD was selected for its reliability and validity when assessing the quality of diverse study designs	QATSDD is the only tool which can be applied to mixed study designs Can be improved with better defined criteria,some items can be clearer by adding examples. Other items could be weighted as more indicative of a rigorous methodology than others (e.g. sample size vs user involvement in design)
Levy I [67].	2017	Israel	Medicine	A systematic review and narrative synthesis investigating the use of complementary medicine for treatment of agitation and delirium in older persons.	QATSDD has been validated in previous studies	We omitted indicator 15 (evidence of user involvement in design),which was determined as irrelevant in a recent comment and critique of this scale (Fenton et.al.,2015)****
Madden C [68].	2018	Ireland	Medicine	A systematic review investigating the potential value of patient record review to assess and improve patient safety in general practice.		The QATSDD has been previously used in other systematic reviews, with high levels of agreement reported. Two reviewers completed the quality assessment and disagreements were resolved through discussion.
Martins-Junior P.A [69].	2017	Brazil	Health Sc	A systematic review investigating dental treatment under general anaesthetic and children’s oral health-related quality of life.		Authors used the Quality Assessment Tool for Studies of Diverse Design (QATSDD) with some modifications to assess the quality of studies. They set up three teams of two investigators to independently extract data for each paper, which lessened subjectivity in article selection and analysis. Also, an inter-reviewer agreement was performed, enhancing the reliability of the results.
McClelland G [70].	2019	UK	Medicine	A narrative review investigating the frequency, characteristics and aetiology of stroke mimic presentations.		A quality assessment tool tailored for cohort studies may have been more appropriate than the QATSDD tool that was chosen before study identification
McPherson A.C [71].	2016	Canada	Medicine	A scoping review investigating best practices when communication with children and families about obesity and weight related topics.		Cut-offs not provided by QATSDD (Low/moderate/high quality ratings)
Medford E [72].	2017	UK	Health Sc	A systematic review investigating the demographic and psychological influences on treatment adherence for children and adolescents with PKU.	QATSDD has shown good reliability and validity and was chosen due to the diverse methodologies of included studies. Inter-rater reliability was good (K = 0.71)	Only studies reporting statistical analyses were included in order to identify the factors most robustly linked with metabolic control and QATSDD was found to be a valid tool for assessing the methodological quality of the studies included in the current review.
Medway M [73].	2016	Australia	Psychology	A qualitative meta-synthesis investigating young people’s experience of family therapy for anorexia nervosa.		Discrepancies in rating resolved by discussion.
Miller L. [74]	2019	Australia	Medicine	A systematic review investigating epidemiology, risk factors and measures for preventing drowning in Africa.		The scores of the criteria were summed up to assess the methodological quality of included studies with a maximum score of 36. For ease of interpretation, the scores were converted to percentages and were categorised as excellent (> 80%), good (50–80%) and low (< 50%) quality of evidence based on the overall score
Mimmo L. [75]	2018	Australia	Health Sc	A systematic review and narrative synthesis investigating patient safety vulnerabilities for children with intellectual disability in hospital.		Disagreements were resolved through discussion resulting in substantial agreement (κ = 0.75) between reviewers on a random sample of 25% of the papers.
Nghiem T [76].	2017	Canada	Medicine		QAT has been validated and found to be reliable for assessing the quality of studies - ICC between pairs of reviewers were 0.840 and 0.703 with CI of 0.776–0.887 and 0.612–0.774,resp.	
Nghiem T [77].	2018	Canada	Medicine	An integrative review investigating pain experience of adults with osteogenesis imperfecta.		Chosen a priori because the tool permits appraisal of studies across a range of designs (i.e., quantitative, qualitative, or mixed method) and would allow the findings to be compared to the review on pain in children and adolescents with Osteogenesis imperfecta.
Noblet T [78].	2017	Sydney	Allied Health	A mixed methods systematic review investigating barriers to and facilitators of independent non-medical prescribing in clinical practice.	Good validity, inter-rater reliability and test-retest reliability have been established for the QATSDD across a variety of study designs, demonstrating its value for consistent quality assessment in mixed methods designs .	-
O’Dowd E [79].	2019	Ireland	Medical Education	A systematic review investigating 7 years of research on entrustable professional activities in graduate medical education from 2011 to 2018.		This assessment tool has been shown to produce good agreement and has been used in a number of different reviews pertaining to health services and medical education research The variability in the quality scores of the development studies is interesting. Although it can be difficult to balance methodological quality and practical success, it is important that researchers developing EPAs give consideration to the quality of their approach. This variability may indicate a need to broadly examine methods in medical education research or to develop methodological quality assessment tools better suited to the field of research.
Orr, K [80].	2019	Canada	Health Sc	A scoping review investigating children and youth with impairments in social skills and cognition in out-of-school time inclusive physical activity programs.		The QATSDD provides a percentage score to compare reporting quality across studies; however, there are no guidelines to suggest values of high or low reporting quality. Thus, based on the guidelines applied in an earlier scoping review (Arbour-Nicitopoulos et al.) the following cut-points were used: less than 60% (low-quality reporting), 60–80% (moderate-quality reporting), and greater than 80% (high-quality reporting).
Pini S [81].	2011	UK	Psychology	A systematic review investigating the effect that cancer diagnosis has on the educational engagement and school life of teenagers.		Absence of official “cut-off ‘’score for quality-assessment.
Powney M [82].	2014	UK	Psychology	A systematic review investigating the attachment and trauma in people with intellectual disabilities.	The QATSDD is a 16-item quality assessment tool, which has shown good inter-rater reliability (k = 71.5%) and validity for the assessment of studies with diverse designs (Sirriyeh, et al., 2012).	For accuracy, the QATSDD was designed to produce an overall quality rating expressed as a percentage.
Quinn C [83].	2018	UK	Medicine	A systematic review investigating the influence of positive aspects of dementia caregiving on caregivers’ wellbeing.		The QATSDD was selected because the items seemed appropriate for the types of papers included; however, there have been criticisms that the QATSDD is too subjective. Although there were clearly benefits in using the QATSDD, there were also challenges to implementing the tool. For instance, we found that studies with smaller word counts (because of journal requirements) risked having a lower score because there is less scope to explain the study in-depth. This suggests that quality-rating tools would benefit from more flexibility: for instance, the ability to take into account the length of the paper.
Rosella L. [84]	2016	Canada	Health Sc	A study investigating the development and validation of a meta-tool for quality appraisal of public health evidence: Meta Quality Appraisal Tool (MetaQAT).		Most tools(including QATSDD) are design-specific and focus only on “risk of bias”. The model of “one tool fits all” approach does not make best use of the existing science of quality appraisal, nor does it adapt in the way articulated in the aforementioned goals**.
Salman Popattia A [85].	2018	Australia	Health Sc	A systematic review investigating the ethical responsibilities of pharmacists when selling complementary medicines.	The tool was selected because it has demonstrated good reliability and validity when applied to a methodologically diverse set of research articles	-
Sibley A.M. [86]	2017	UK	Health Sc	A mixed-methods study investigation diabetes patients’ beliefs about their medicines from a nurse prescribers’ perspective.	The ‘quality assessment tool’ reported inter-rater reliability as ‘substantial’ to ‘very substantial’ (kappa ranging from 0.69 to 0.91) for question level agreement (Sirriyeh et al. 2012).	-
Ten Hoorn S [87].	2016	Netherlands	Medicine	A systematic review investigating communication with conscious and mechanically ventilated critically ill patients.	Test-retest and inter-rater reliabilities range from “good” to “substantial”(K 0.698–0.901).	Can be applied to diverse study designs Clearly defined scales
Tomlin E.M [88].	2018	UK	Psychology	A thesis investigating the experience-based co-design approach within the NHS with patients at the centre of design to improve quality of care.	This validated tool has demonstrated good internal reliability and is seen as a pragmatic approach to providing a robust, transparent and standardized method to assess quality across different research methodologies.	Owing to the heterogeneity of study designs included within the inclusion criteria, the Quality Assessment Tool for Studies with Diverse Designs (QATSDD) was initially pre-specified as an appropriate method to assess study quality. The criteria did not map onto the reported content of the EBCD QI projects. This meant that relevant data was not accurately assessed and ultimately made the comparison between the EBCD QI projects and evaluative and research papers problematic. For instance: evidence of sample size considered in terms of analysis, representative sample of target group of a reasonable size and fit between research questions and method of analysis were not considered to be routinely reported aspects of EBCD QI projects. Therefore, a dual approach was taken to assess the quality of included papers within the review.
Tuominen O [89].	2018	Australia, Finland	Health Sc	A scoping review investigating the rescheduling of nursing staff with information technology-based staffing solutions.	Agreement of scoring between the two reviewers was calculated using a Cohen’s kappa. Kappa values varied between the two reviewers from 0.78 to 1.00 for these ten papers, showing good (0.60 to 0.80) or very good (> 0.80 to 1.00) agreement.	-
Vyth E.L. [90]	2012	Netherlands	Allied Health	A review investigating the methodological quality of front-of-pack labelling studies.	Initial coder disagreement of 55 of the 496 scores, resolved completely after discussion.	Applicable to diverse research designs.
Wallace A [91].	2016	UK	Medicine	A systematic review investigating the traumatic dental injury research.	Inter examiner agreement for QA was poorer (54–82%)than for general data-extraction; Intra-examiner agreement after 8-weeks interval was good for QA (64–76%)	- QATSDD is a unique quality assessment tool can be applied to diverse study designs, unlike majority of other tools which evaluate a single methodological approach - No authors referred to an explicit theoretical framework or model to underpin their work. This is a difficult concept and may not have been relevant to all study designs. Indeed, the developers of the QATSDD acknowledge that some of quality criteria may not be suitable for certain study designs. Theoretical frameworks may be more appropriate to studies with a qualitative component, and they may help to inform the study design and explain findings. The absence of any qualitative studies therefore makes the results less remarkable. - Standard deviation for the scores ascribed to studies in the quality assessment exercise was high (18.7%). One explanation for this may be a true marked difference in quality of the papers. Characteristics of the QATSDD rather than the study quality per se may explain the low scores for some papers, and hence the high standard deviation. - The QATSDD uses a 4‐point scoring system to try and provide a more accurate representation of the paper’s quality. However, this scaled scoring system means there is a wider margin for disagreement between reviewers compared to a 2‐point scoring scale - The research experience of the reviewers in this present review was quite varied and may account for the inconsistencies in inter‐ and intra‐examiner agreement. Furthermore, it was felt that the QATSDD would benefit from the provision of greater detail in some of the descriptions to improve inter-examiner agreement. However, the QATSDD was found to be generally applicable to the range of study designs encountered in this review.
Walton M [92].	2015	Australia	Health Sc	A systemtatic review and narrative sysnthesis investigating workplace training for senior trainees.	Substantial agreement (k = 62.5%)confirmed between reviewers	
Wells, E [93].	2016	UK	Health Sc	A thesis study investigating the role of parenting interventions in promoting treatment adherence in cystic fibrosis.	An independent researcher rated 7 of the 15 included papers (47%) and any discrepancies were resolved through discussion. Cohen’s Kappa was 0.71 indicating ‘substantial’ interrater agreement.	-
Wright C.J [94].	2017	Australia	Philosophy		The QATSDD has adequate face validity, inter-rater reliability (κ = 71.5%; indicating substantial agreement), and good to substantial agreement for test-retest reliability	Although the tool has been empirically tested, concerns have been raised regarding the use of scales (i.e., thought to affect the establishment of inter-rater reliability) and its ease of application

### Excluded studies

Reasons for exclusion of studies were that 97 had cited the paper but made no further comments, 38 had cited the tool and provided rationale for its selection as the preferred tool but did not produce reliability and validity data, and 21 produced reliability and validity data that confirmed consistently the tool was reliable and valid across multiple contexts but made no qualitative comments. A further two papers were incorrectly attributed to the article on Google Scholar and eight were non-English papers.

### Findings regarding the QATSDD tool

The synthesis revealed a number of perceived areas of strength of the tool including its strong reliability and validity [33, 55, 66]. All of the reviews within the 39 included articles that used the tool confirmed its reliability and validity. Further strength were the ability of QuADS to be applied when appraising diverse study designs [66, 95, 96], and its comprehensive list of indicators [97]. The breadth of disciplines in which this tool had been applied was notable: psychology, medicine, health sciences, allied health, and health services. The final group of included studies reflected the range of disciplines in which the tool had been applied. Authors who had employed the tool commented that it was valued for its inclusion of a wider range of important issues relating to research quality such as the involvement of end users in the research design and process, facilitating a comprehensive analysis [31, 95] Further, the synthesis also revealed opportunities to clarify and improve the tool, with one study [12] that conducted a substantial commentary piece on the QATSDD tool and its applications. Five key areas in which there were opportunities for enhancements or further clarification emerged. A number of revisions were made as part of the present study to the tool in order to address the findings from this study and described in relation to each of the findings below.

#### Scope and purpose of the tool

Further clarification on the scope of the use of the tool appeared to be necessary to distinguish its focus predominantly on reviews of mixed- or multi-method studies but also its purpose in providing an approach to assess the transparency and quality of study reporting. Instances in which the tool had been applied to exclude studies from a review were noted and this appeared to be due to the lack of detail regarding the purpose of the tool available to reviewers. Lack of clarity regarding the method for scoring using the QATSDD was apparent, with queries including whether weighting was required for particular criteria and the need for a cut-off to delineate high and low quality studies [12, 55, 91, 98]. Such queries indicate that the purpose of the tool to stimulate discussion regarding the quality and transparency of reporting in relation to each study may not be clear. There is no evidence to suggest that any criterion is more important than another or that a particular score is indicative of high or low quality; therefore, any cut-off would be arbitrary. The tool enables researchers to consider and discuss each element of the study in the context of its research aims and to explore the extent to which each quality criterion is met. This may then stimulate discussion of its relative importance in the context of their own review. A summary of the purpose of the tool and its scope is included in a new ‘User guide’ (supplementary file 1) that accompanies the tool.

#### Examples for each criterion

The desire for more examples to be used as part of the tool’s criteria was highlighted by Fenton et al. [12] and Lamore et al. [66]. These papers provided commentary that the use of more explicit examples from both a quantitative and qualitative perspective would assist users when scoring. These authors found the tool challenging when examples did not match the methods used in the papers they were reviewing and highlighted an opportunity to be more inclusive of a wider range of possible research methodologies when providing examples. Furthermore, the inclusion of additional examples may address challenges of distinguishing between scores. Limiting the responses to a dichotomous scale or 3-point scoring system was suggested in one commentary but a dichotomous scale does not provide sufficient response options for many items that are more complex than a yes or no, and three-point scales are recognised as leading to the overuse of neutral responses [12].

#### Theoretical and conceptual framework

A common challenge identified was in applying the notion of a ‘theoretical framework,’ particularly outside the discipline of Psychology [12, 91]. Fenton et al. [12] highlighted the need for additional guidance regarding the a definition of a theoretical framework and specifically, whether the inclusion of reference to theoretical concepts or assumptions was relevant to this criterion. It was notable from the included reviews that few studies scored highly on this criterion, providing a further indicator that this may require review. To resolve this, the criterion ‘Theoretical framework’ was revised to ‘Explicit consideration of theories or concepts that frame the study in the introduction,’ with relevant exemplars.

#### Quantitative bias, appropriate sampling and analytic methods

Fenton et al. indicated that the tool held a quantitative bias [12], suggesting that the wording and selection of examples may favour quantitative studies. Clausen et al. [33] also suggested that qualitative studies performed poorly using the tool. Criteria related to appropriate sampling and analytic methods appeared to be challenging to assess and it was decided to update these in the light of current perspectives on qualitative methodology, particularly regarding matters such as the need (or lack of) for data saturation. Explicit examples and language were added to each descriptor to balance recognition of both qualitative and quantitative research. Criteria concerning sample sizes was revised and reduced to ‘Appropriate sampling to address the research aim/s.’

## Discussion

Quality appraisal is both a widely-debated and dynamic area with emerging opportunities but also increasing demands [98]. The findings of this research show that the QATSDD tool was utilised in a wide variety of health fields including psychology, allied health, medicine, public health, nursing, health services and social sciences, and that the tool demonstrated high reliability. Nevertheless, a range of minor limitations regarding the scope of use of the tool, balance between qualitative and quantitative ontologies and ease of use through examples also came to light. In the context of increasing mixed and multi-methods research in health services, this paper has described the development of the QuADS tool which is an augmentation of the QATSDD, and aims to be one of few pragmatic tools that will enable quality assessment across a diverse range of study designs [99]. QuADS provides a basis for research teams to reflect on methodological and evidence quality, in addition to establish limitations in the quality of reporting of studies. There is complementary scope for application of QuADS with other tools that focused on appraising the methodological quality to provide an expanded analysis where needed [100].

Increasing recognition of the value of employing mixed methods approaches in health services research to address complex healthcare questions is reflected in more than 10 quality assessment methods for mixed-methods work [101]. Such approaches have focused to the justification for and application of mixed methods in the study, considering approaches to study design and data synthesis. Current methods to explore quality in mixed-methods studies may not readily apply in the context of multi-methods work or a collection of heterogenous studies in a systematic review [101]. Given the multitude of quantitative or qualitative quality appraisal tools, a segregated approach is often taken to explore quality when reviews include heterogeneous studies which limits researcher ability to comment on the body of evidence collectively.

Four tools, including the QATSDD, have been developed to date to enable an integrated quality assessment [13, 102–104]. Two of the available tools provide a segregated analysis of the qualitative and quantitative elements of research studies rather than a single set of items applicable to explore both [102, 104]. The remaining tool provides a method to explore completeness of reporting of studies with mixed or multiple methods [103]. In the context of existing tools, the QuADS enables a brief, integrated assessment to be undertaken across a body of evidence within a review.

### Limitations

This manuscript reports the first stage in revising a pragmatic tool that can be used to help guide reporting of research and to make assessments of the quality of non-trial based mixed- and multi-methods studies. Methodological, evidence and reporting quality are three important areas and each complex in their own right. Addressing all of these elements with a single tool is valuable for stimulating discussion and reflection between reviewers but provides a high-level analysis of these different quality domains. Ultimately the tool does not therefore provide a conclusive outcome regarding the quality of the research that can be used to make decisions regarding the inclusion or exclusion of studies from a review. Despite the inclusion of a wide range of literature utilising the QATSDD tool, the response rate of authors in the survey component of this work was very low which may have shaped the information provided. This project benefited from drawing upon the insights of those who had utilised the tool to shape the design of the revised tool, yet it is possible that those experienced difficulty in using the QATSDD tool ultimately did not include the tool in their outputs and were not readily identifiable for inclusion in this project. As a result, we may not have identified all of the areas for refinement required. Whilst the study panel were all experienced in reporting studies with diverse designs in multiple locations internationally, the panel process did not constitute a formal Delphi approach required to register QuADS tool in the Equator Network as a reporting guideline. This further process is an important further subsequent step that we seek to complete to improve the rigour and evidence base for the new tool.

## Conclusion

Quality appraisal continues to be a critical component of systematic review. Increasing recognition of the value of multi- and mixed methods research to address complex health services research questions requires a tool such as QuADS, demonstrating good reliability and which allows researchers to appraise heterogenous studies in systematic review.

## Supplementary Information

**Additional file 1.**

**Additional file 2.** QuADS Criteria.

## Data Availability

Data may be requested by contacting the first author.

## Abbreviations

QATSDD: Quality Assessment Tool for Studies with Diverse Designs
QuADS: Quality Assessment for Diverse Studies (QuADS) tool

## References

[1] Booth A (2016). Searching for qualitative research for inclusion in systematic reviews: a structured methodological review. Syst Rev.

[2] Dixon-Woods M (2006). How can systematic reviews incorporate qualitative research? A critical perspective. Qual Res.

[3] Dixon-Woods M, Fitzpatrick R (2001). Qualitative research in systematic reviews: has established a place for itself. Br Med J.

[4] Collins KM, Onwuegbuzie AJ, Sutton IL (2006). A model incorporating the rationale and purpose for conducting mixed methods research in special education and beyond. Learn Disabil Contemp J.

[5] Morse JM. Mixed method design: principles and procedures: Routledge; 2016.

[6] Verhagen AP (2001). The art of quality assessment of RCTs included in systematic reviews. J Clin Epidemiol.

[7] Dixon-Woods M (2004). The problem of appraising qualitative research. BMJ Qual Safety.

[8] Carroll C, Booth A (2015). Quality assessment of qualitative evidence for systematic review and synthesis: is it meaningful, and if so, how should it be performed?. Res Synth Methods.

[9] Dixon-Woods M (2007). Appraising qualitative research for inclusion in systematic reviews: a quantitative and qualitative comparison of three methods. J Health Serv Res Policy.

[10] Hannes K, Macaitis K (2012). A move to more systematic and transparent approaches in qualitative evidence synthesis: update on a review of published papers. Qual Res.

[11] Kmet LM, Cook LS, Lee RC (2004). Standard quality assessment criteria for evaluating primary research papers from a variety of fields.

[12] Fenton L, Lauckner H, Gilbert R (2015). The QATSDD critical appraisal tool: comments and critiques. J Eval Clin Pract.

[13] Sirriyeh R (2012). Reviewing studies with diverse designs: the development and evaluation of a new tool. J Eval Clin Pract.

[14] Popay J (2006). Guidance on the conduct of narrative synthesis in systematic reviews. A product from the ESRC methods programme.

[15] Chauhan A (2020). The safety of health care for ethnic minority patients: a systematic review. Int J Equity Health.

[16] Abda A, Bolduc ME, Tsimicalis A, Rennick J, Vatcher D, Brossard-Racine M (2019). Psychosocial outcomes of children and adolescents with severe congenital heart defect: a systematic review and meta-analysis. J Pediatr Psychol.

[17] Adam A, Jensen JD (2016). What is the effectiveness of obesity related interventions at retail grocery stores and supermarkets?—a systematic review. BMC Public Health.

[18] Albutt AK, O'Hara JK, Conner MT, Fletcher SJ, Lawton RJ (2017). Is there a role for patients and their relatives in escalating clinical deterioration in hospital? A systematic review. Health Expect.

[19] Alsawy S, Mansell W, McEvoy P, Tai S (2017). What is good communication for people living with dementia? A mixed-methods systematic review. Int Psychogeriatr.

[20] Arbour-Nicitopoulos KP, Grassmann V, Orr K, McPherson AC, Faulkner GE, Wright FVA (2018). Scoping review of inclusive out-of-school time physical activity programs for children and youth with physical disabilities. Adapt Phys Act Q.

[21] Augestad LB. Self-concept and self-esteem among children and young adults with visual impairment: a systematic review. Cogent Psychol. 2017;4.

[22] Augestad LB (2017). Mental health among children and young adults with visual impairments: a systematic review. J Vis Impairment Blindness.

[23] Aztlan-James EA, McLemore M, Taylor D (2017). Multiple unintended pregnancies in US women: a systematic review. Womens Health Issues.

[24] Band R, Wearden A, Barrowclough C (2015). Patient outcomes in association with significant other responses to chronic fatigue syndrome: a systematic review of the literature. Clin Psychol Sci Pract.

[25] Batten G, Oakes PM, Alexander T (2014). Factors associated with social interactions between deaf children and their hearing peers: a systematic literature review. J Deaf Stud Deaf Educ.

[26] Baxter R, Taylor N, Kellar I, Lawton R (2016). What methods are used to apply positive deviance within healthcare organisations? A systematic review. BMJ Qual Saf.

[27] Blackwell JE, Alammar HA, Weighall AR, Kellar I, Nash HM (2017). A systematic review of cognitive function and psychosocial well-being in school-age children with narcolepsy. Sleep Med Rev.

[28] Blake DF, Crowe M, Mitchell SJ, Aitken P, Pollock NW (2018). Vibration and bubbles: a systematic review of the effects of helicopter retrieval on injured divers. Diving Hyperb Med.

[29] Bradford N, Chambers S, Hudson A (2019). Evaluation frameworks in health services: an integrative review of use, attributes and elements. J Clin Nurs.

[30] Braun SE, Kinser PA, Rybarczyk B (2019). Can mindfulness in health care professionals improve patient care? An integrative review and proposed model. Transl Behav Med.

[31] Burton A (2017). How effective are mindfulness-based interventions for reducing stress among healthcare professionals? A systematic review and meta-analysis. Stress Health.

[32] Carrara A, Schulz PJ (2018). The role of health literacy in predicting adherence to nutritional recommendations: a systematic review. Patient Educ Couns.

[33] Clausen C, Cummins K, Dionne K (2017). Educational interventions to enhance competencies for interprofessional collaboration among nurse and physician managers: an integrative review. J Interprof Care.

[34] Connolly F, Byrne D, Lydon S, Walsh C, O’Connor P (2017). Barriers and facilitators related to the implementation of a physiological track and trigger system: a systematic review of the qualitative evidence. Int J Qual Health Care.

[35] Curran C, Lydon S, Kelly M, Murphy A, Walsh C, O’Connor P. A systematic review of primary care safety climate survey instruments: their origins, psychometric properties, quality, and usage. J Patient Saf 2018;14(2):e9–18. 10.1097/PTS.0000000000000393 28708671

[36] Deming A, Jennings JL. The absence of evidence-based practices (EBPs) in the treatment of sexual abusers: recommendations for moving toward the use of a true EBP model. Sex Abus. 2019:1079063219843897. 10.1177/1079063219843897 31010394

[37] Dias CC, Rodrigues PP, da Costa-Pereira A, Magro F (2013). Clinical prognostic factors for disabling Crohn's disease: a systematic review and meta-analysis. World J Gastroenterol: WJG.

[38] Emerson LM, Leyland A, Hudson K, Rowse G, Hanley P, Hugh-Jones S (2017). Teaching mindfulness to teachers: a systematic review and narrative synthesis. Mindfulness..

[39] Fenton L, White C, Gallant K, Hutchinson S, Hamilton-Hinch B, Gilbert R, Lauckner H. The benefits of recreation for the recovery and social inclusion of individuals with mental health challenges: An integrative review.

[40] Filmer T, Herbig B (2018). Effectiveness of interventions teaching cross-cultural competencies to health-related professionals with work experience: a systematic review. J Contin Educ Health Prof.

[41] Fylan B. Medicines management after hospital discharge: patients’ personal and professional networks (Doctoral dissertation, University of Bradford).

[42] Graham-Clarke E, Rushton A, Noblet T, Marriott J (2018). Facilitators and barriers to non-medical prescribing–a systematic review and thematic synthesis. PLoS One.

[43] Gillham R, Wittkowski A (2015). Outcomes for women admitted to a mother and baby unit: a systematic review. Int J Women’s Health.

[44] Gkika S, Wittkowski A, Wells A (2018). Social cognition and metacognition in social anxiety: a systematic review. Clin Psychol Psychother.

[45] Hardy M, Johnson L, Sharples R, Boynes S, Irving D (2016). Does radiography advanced practice improve patient outcomes and health service quality? A systematic review. Br J Radiol.

[46] Harris K, Band RJ, Cooper H, Macintyre VG, Mejia A, Wearden AJ (2016). Distress in significant others of patients with chronic fatigue syndrome: a systematic review of the literature. Br J Health Psychol.

[47] Harrison R, Cohen AW, Walton M (2015). Patient safety and quality of care in developing countries in Southeast Asia: a systematic literature review. Int J Qual Health Care.

[48] Harrison R, Walton M, Manias E, Smith-Merry J, Kelly P, Iedema R, Robinson L (2015). The missing evidence: a systematic review of patients’ experiences of adverse events in health care. Int J Qual Health Care.

[49] Harrison R, Birks Y, Hall J, Bosanquet K, Harden M, Iedema R (2014). The contribution of nurses to incident disclosure: a narrative review. Int J Nurs Stud.

[50] Hawkins RD. Psychological factors underpinning child-animal relationships and preventing animal cruelty (Doctoral dissertation, University of Edinburgh).

[51] Heath G, Montgomery H, Eyre C, Cummins C, Pattison H, Shaw R (2016). Developing a tool to support communication of parental concerns when a child is in hospital. InHealthcare.

[52] Hesselstrand M, Samuelsson K, Liedberg G (2015). Occupational therapy interventions in chronic pain–a systematic review. Occup Ther Int.

[53] Hill S, Adams J, Hislop J (2015). Conducting contingent valuation studies in older and young populations: a rapid review.

[54] Holl M, van den Dries L, Wolf JR. Interventions to prevent tenant evictions: a systematic review. Health Soc Care Community,. 2016;24(5):532–546. 10.1111/hsc.12257 26109137

[55] Iddon J, Dickson J, Unwin J (2016). Positive psychological interventions and chronic non-cancer pain: a systematic review of the literature. Int J Appl Positive Psychol.

[56] Jaarsma EA, Smith B (2018). Promoting physical activity for disabled people who are ready to become physically active: a systematic review. Psychol Sport Exerc.

[57] Jackman PC, Hawkins RM, Crust L, Swann C (2019). Flow states in exercise: a systematic review. Psychol Sport Exerc.

[58] Jackson-Blott K, Hare D, Davies B, Morgan S (2019). Recovery-oriented training programmes for mental health professionals: a narrative literature review. Ment Health Prev.

[59] Johnson D, Horton E, Mulcahy R, Foth M (2017). Gamification and serious games within the domain of domestic energy consumption: a systematic review. Renew Sust Energ Rev.

[60] Jones N, Bartlett H (2018). The impact of visual impairment on nutritional status: a systematic review. Br J Vis Impair.

[61] Khajehaminian MR, Ardalan A, Keshtkar A (2018). A systematic literature review of criteria and models for casualty distribution in trauma related mass casualty incidents. Injury.

[62] Klingenberg O, Holkesvik AH, Augestad LB. Digital learning in mathematics for students with severe visual impairment: a systematic review. Br J Vis Impair. 2019;00(0).

[63] Kolbe AR. ‘It’s not a gift when it comes with price’: a qualitative study of transactional sex between UN peacekeepers and Haitian citizens. Stability Int J Secur Dev. 2015.

[64] Kumar MB, Wesche S, McGuire C (2012). Trends in metis-related health research (1980–2009): identification of research gaps. Can J Public Health.

[65] Lambe KA, Lydon S, Madden C (2019). Hand hygiene compliance in the ICU: a systematic review. Crit Care Med.

[66] Lamore K, Montalescot L, Untas A (2017). Treatment decision-making in chronic diseases: what are the family members’ roles, needs and attitudes? A systematic review. Patient Educ Couns.

[67] Levy I, Attias S, Ben-Arye E, Bloch B, Schiff E (2017). Complementary medicine for treatment of agitation and delirium in older persons: a systematic review and narrative synthesis. Int J Geriatr Psychiatry.

[68] Madden C, Lydon S, Curran C, Murphy AW, O’Connor P (2018). Potential value of patient record review to assess and improve patient safety in general practice: a systematic review. Eur J Gen Pract.

[69] Martins-Junior PA (2017). Dental treatment under general anaesthetic and children’s oral health-related quality of life. Evid Based Dent.

[70] McClelland G, Rodgers H, Flynn D, Price CI (2019). The frequency, characteristics and aetiology of stroke mimic presentations: a narrative review. Eur J Emerg Med.

[71] McPherson AC, Hamilton J, Kingsnorth S (2017). Communicating with children and families about obesity and weight-related topics: a scoping review of best practices. Obes Rev.

[72] Medford E, Hare DJ, Wittkowski A (2017). Demographic and psychosocial influences on treatment adherence for children and adolescents with PKU: a systematic review. JIMD Rep.

[73] Medway M, Rhodes P (2016). Young people’s experience of family therapy for anorexia nervosa: a qualitative meta-synthesis. Adv Eat Disord.

[74] Miller L, Alele FO, Emeto TI, Franklin RC. Epidemiology, risk factors and measures for preventing drowning in Africa: a systematic review. Medicina. 2019;55(10). 10.3390/medicina55100637 PMC6843779 31557943

[75] Mimmo L, Harrison R, Hinchcliff R. Patient safety vulnerabilities for children with intellectual disability in hospital: a systematic review and narrative synthesis. BMJ Paediatr Open. 2018;2(1). 10.1136/bmjpo-2017-000201 PMC5843001 29637187

[76] Nghiem T, Louli J, Treherne SC, Anderson CE, Tsimicalis A, Lalloo C, Stinson JN, Thorstad K (2017). Pain experiences of children and adolescents with osteogenesis imperfecta. Clin J Pain.

[77] Nghiem T, Chougui K, Michalovic A, Lalloo C, Stinson J, Lafrance ME, Palomo T, Dahan-Oliel N, Tsimicalis A (2018). Pain experiences of adults with osteogenesis imperfecta: an integrative review. Can J Pain.

[78] Noblet T, Marriott J, Graham-Clarke E, Rushton A (2017). Barriers to and facilitators of independent non-medical prescribing in clinical practice: a mixed-methods systematic review. J Phys.

[79] O'Dowd E, Lydon S, O'Connor P, Madden C, Byrne D (2019). A systematic review of 7 years of research on entrustable professional activities in graduate medical education, 2011-2018. Med Educ.

[80] Orr K, Wright FV, Grassmann V, McPherson AC, Faulkner GE, Arbour-Nicitopoulos KP. Children and youth with impairments in social skills and cognition in out-of-school time inclusive physical activity programs: a scoping review. Int J Dev Disabil. 2019. 10.1080/20473869.2019.1603731 PMC8115467 34141401

[81] Pini S, Hugh-Jones S, Gardner PH (2012). What effect does a cancer diagnosis have on the educational engagement and school life of teenagers? A systematic review. Psycho-Oncology.

[82] Powney M (2014). Attachment and trauma in people with intellectual disabilities.

[83] Quinn C, Toms G. Influence of positive aspects of dementia caregiving on caregivers’ well-being: a systematic review. The Gerontologist. 2018. 10.1093/geront/gny168 30597058

[84] Rosella L, Bowman C, Pach B, Morgan S, Fitzpatrick T, Goel V (2016). The development and validation of a meta-tool for quality appraisal of public health evidence: meta quality appraisal tool (MetaQAT). Public Health.

[85] Salman Popattia A, Winch S, La Caze A (2018). Ethical responsibilities of pharmacists when selling complementary medicines: a systematic review. Int J Pharm Pract.

[86] Sibley A. Nurse prescribers’ exploration of diabetes patients’ beliefs about their medicines (Doctoral dissertation, University of Southampton).

[87] Ten Hoorn S, Elbers PW, Girbes AR, Tuinman PR (2016). Communicating with conscious and mechanically ventilated critically ill patients: a systematic review. Crit Care.

[88] Tomlin M. Patients at the centre of design to improve the quality of care; exploring the experience-based co-design approach within the NHS: [Doctor of Philosophy]: School of Psychology, The University of Leeds; 2018.

[89] Tuominen O, Lundgrén-Laine H, Flinkman M, Boucht S, Salanterä S (2018). Rescheduling nursing staff with information technology-based staffing solutions: a scoping review. Int J Healthc Technol Manag.

[90] Vyth EL, Steenhuis IH, Brandt HE, Roodenburg AJ, Brug J, Seidell JC (2012). Methodological quality of front-of-pack labeling studies: a review plus identification of research challenges. Nutr Rev.

[91] Wallace A (2017). Traumatic dental injury research: on children or with children?. Dent Traumatol.

[92] Walton M, Harrison R, Burgess A, Foster K (2015). Workplace training for senior trainees: a systematic review and narrative synthesis of current approaches to promote patient safety. Postgrad Med J.

[93] Wells E (2016). The role of parenting interventions in promoting treatment adherence in cystic fibrosis.

[94] Wright CJ. Likes, dislikes, must-haves, and must-nots: an exploratory study into the housing preferences of adults with neurological disability: School of Human Services and Social Work, Griffith University; 2017.

[95] Arbour-Nicitopoulos KP (2018). A scoping review of inclusive out-of-school time physical activity programs for children and youth with physical disabilities. Adapt Phys Act Q.

[96] Noblet T (2017). Barriers to and facilitators of independent non-medical prescribing in clinical practice: a mixed-methods systematic review. J Physiother.

[97] Tomlin M (2018). Patients at the centre of design to improve the quality of care; exploring the experience-based co-design approach within the NHS, in School of Psychology.

[98] Harrison JK (2017). Using quality assessment tools to critically appraise ageing research: a guide for clinicians. Age Ageing.

[99] Hong QN (2019). Improving the content validity of the mixed methods appraisal tool: a modified e-Delphi study. J Clin Epidemiol.

[100] Hong QN, Sergic F, Bartlett G, Boardman F, Cargo M, Dagenais P, Gagnon MP, Griffiths F, Nicolau B, O’Cathain A, Rousseau M-C, Vedel I, Pluye P. The mixed methods appraisal tool (MMAT) version 2018 for information professionals and researchers. Educ Inf. 2018;34(4):285–91.

[101] Curry L, NSM (2015). Mixed methods in health sciences research: a practical primer.

[102] AFL (2005). Evaluative tool for mixed method studies. Schools of healthcare.

[103] Crowe M, Sheppard L, Campbell A (2012). Reliability analysis for a proposed critical appraisal tool demonstrated value for diverse research designs. J Clin Epidemiol.

[104] Pace R (2012). Testing the reliability and efficiency of the pilot mixed methods appraisal tool (MMAT) for systematic mixed studies review. Int J Nurs Stud.

