# Correction to: Quality assessment with diverse studies (QuADS): an appraisal tool for methodological and reporting quality in systematic reviews of mixed- or multimethod studies

**DOI:** 10.1186/s12913-021-06261-2

**Published:** 2021-03-16

**Authors:** Reema Harrison, Benjamin Jones, Peter Gardner, Rebecca Lawton

**Affiliations:** 1 School of Population Health, UNSW Sydney, Sydney, Australia; 2 School of Pharmacy and Medical Sciences, University of Bradford, Bradford, UK; 3 Institute of Psychological Sciences, University of Leeds, Leeds, UK

**Correction to: BMC Health Serv Res (2021) 21:144**

**https://doi.org/10.1186/s12913-021-06122-y**

Following publication of the original article [1], the authors identified an error in the author name of Peter Gardner.

The incorrect author name is: Peter Gardener.

The correct author name is: Peter Gardner.

The author group has been updated above and the original article [1] has been corrected.
