# Supplementary material for: Quality assessment with diverse studies (QuADS): an appraisal tool for methodological and reporting quality in systematic reviews of mixed- or multi-method studies
Source: BMC Health Serv Res. 2021 Feb 15;21:144. doi: 10.1186/s12913-021-06122-y (PMC7885606; doi:10.1186/s12913-021-06122-y)
Supplement: Supplementary file 1 — Additional file 1. [file 12913_2021_6122_MOESM1_ESM.docx]

Quality Assessment for Diverse Studies (QuADS): User Guide v1.0

**Step 1: Establish whether QuADS is relevant for your review**

The QuADS tool is developed for use in literature reviews that include multi-method or mixed-methods research. The tool is applied to the studies that are selected for inclusion in the review based on the eligibility criteria. Whilst the tool can be applied to reviews that have a group of quantitative and a group of qualitative studies within, it is also possible to assess each group separately and then conduct a synthesised quality assessment as an alternate approach. It is important to consider the relevance of each of the available tools to your group of papers to determine which approach will allow you to optimally assess the body of work. The QuADS tool can also be used with reviews of multi- or mixed methods work and some examples are given as to how the criteria could be applied in the context of a review article.

**Step 2: Agree the way in which your team will apply the QuADS criteria**

Once the review team have finalised the studies to be included in the review, the team should first discuss the way in which the tool will be applied to the resultant studies. The QuADS tool requires reviewers to apply their own research knowledge and judgement in making decisions around the appropriate scoring for each study. An optimal process occurs when the reviewer ream collectively discuss the QuADS criteria and how they might apply these in the context of the body of work of focus. The facilitates reviewers to develop a shared understanding of the items in the context of their review.

**Step 3: Independently apply the QuADS criteria to a small group of included studies**

Exploring the quality of research reporting and conduct requires an iterative process to enable the review team to consolidate a shared understanding of what high quality reporting looks like in their given field or research topic. The team members should independently try to apply the QuADS tool to the same 3-5 articles. The scores are applied on a four-point scale to enable researchers to distinguish the degree to which a criteria is met.

**Step 4: Instigate reviewer discussion**

Once the criteria have been applied to a small number of studies, the review team can then meet to discuss their scoring and experiences. Through this process, the team will finalise a shared understanding of how they will apply these criteria and discuss any inconsistencies or challenges in application of the criteria to particular studies or the group of studies.

**Step 5: Independently apply the QuADS criteria to a further group of included studies or the remainder**

Once agreement around the application of the tool to the work has been reached through steps 3 and 4, each reviewer can then apply the criteria independently to all studies in the review, or this may be a subset in the context of reviews of a high volume of papers. In the context of substantial disagreement or difficulty in applying the criteria to any given study or body of work, it may be necessary to repeat steps 3 and 4 to consolidate an understanding of how to apply the tool to the given body of work.

**Step 6: Review the scores obtained across the body of work against each criteria**

Once all papers have been reviewed by all reviewers, the team once again meet to discuss their scoring, any discrepancies and those discrepancies that may be resolved. The reviewers will finalise their scores and may then wish to undertake an inter-rater reliability analysis with these.

**Step 7: Using the resulting data**

The resulting data from the QuADS tool provides researchers an opportunity to consider the reporting of information across the body of work related to each criterion. Criteria are not weighted but some may be considered by the research team to be particularly important to their research field and these findings can be discussed narratively when writing up findings regarding the study quality assessment.

There is also no cut-off score for a study to be considered high or low quality; any cut-off would be arbitrary and not appropriate when using this tool. Through reviewing scores for each single criterion across the body of work rather than the overall scores, researchers are advised to discuss the quality assessment findings narratively and consider areas in which reporting is comprehensive or less so and why this may be.
